# Experiences of LGBTIQA+ People with Disability in Healthcare and Community Services: Towards Embracing Multiple Identities

**DOI:** 10.3390/ijerph17218080

**Published:** 2020-11-02

**Authors:** Amie O’Shea, J. R. Latham, Ruth McNair, Nathan Despott, Mellem Rose, Ruby Mountford, Patsie Frawley

**Affiliations:** 1 School of Health & Social Development, Deakin University, Geelong 3217, Australia; mellem.rose@gmail.com (M.R.); ruby.mountford@deakin.edu.au (R.M.); 2 Alfred Deakin Institute for Citizenship and Globalisation, Deakin University, Burwood 3125, Australia; textual.gore@gmail.com; 3 School of Culture and Communication, University of Melbourne, Parkville 3010, Australia; 4 Department of General Practice, University of Melbourne, Parkville 3010, Australia; r.mcnair@unimelb.edu.au; 5 Inclusion Melbourne, Armadale 3143, Australia; nathan.despott@inclusion.melbourne; 6 Te Kura Toi Tangata School of Education, University of Waikato, Hamilton 3216, New Zealand

**Keywords:** disability, sexuality, gender, LGBTIQA+, identity, service provision, LGBT+ health equity, health inequalities

## Abstract

Healthcare and disability support services are increasing their efforts towards inclusion and recognising the needs of different groups. This research project was conducted by academic and peer researchers (LGBTIQA+ people with disability) in Victoria, Australia using four focus groups with LGBTIQA+ people with disability. We report on two overarching themes relating to participants’ experiences of accessing health services as LGBTIQA+ people with disability: difficulties in managing multiple identities and the impacts of community services and supports. Participants described having to repeatedly ‘come out’ in a range of ways and contexts as complex and layered processes in which it was difficult to present their full range of needs and experiences to services. We also found that the role of community in promoting a sense of belonging and resilience increased capacity to manage health service use and advocacy. Services and communities aiming to be inclusive to all have the opportunity to recognise and respond to the issues faced by LGBTIQA+ people with disability as a way to pay attention to how overt and subtle practices of discrimination continue to operate despite repeated attempts at or claims of being ‘inclusive.’ Our research suggests actual inclusive, accessible services can be achieved in part through policy and practice that actively responds to the specific needs of LGBTIQA+ people with disability, in addition to LGBTIQA+ education for disability services and disability and accessibility education for LGBTIQA+ focused services. As we do in this article, we argue that this work must be done by prioritising authentic participation of LGBTIQA+ people with disability in the services and research that is about them.

## 1. Introduction

In recent years, political and healthcare systems have become more engaged with seeking to provide appropriate care for LGBTIQA+ people, along with other marginalised categories of people, to reconsider inclusive practice [1,2]. The acronym LGBTIQA+ stands for lesbian, gay, bisexual, transgender (or trans*), intersex, queer/questioning, asexual, HIV positive and other terms (such as non-binary and pansexual) that people use to describe or express their sex, gender, sexuality, intersex status and relationships. Intersex people are born with physical sex characteristics that do not fit medical norms for female or male bodies. The term ‘queer’ is often used as an umbrella term to refer to sexually and gender diverse people. Although historically used as a pejorative, many LGBTIQA+ people have reclaimed the term as an expression of resistance, solidarity and sense of belonging to a broad community. As with many identity categories, the language used to describe these experiences is constantly changing. There is no one preferred term; people often have multiple, overlapping identities, and many people and communities also have unique ways of describing their identities, histories and experiences. We use LGBTIQA+ as a collective term to describe people who see themselves included by its use. 

LGBTIQA+ people have the right to health services that overtly cater to their specific needs [3,4]. Initial moves towards this involved encouraging LGBTIQA+ people to disclose to healthcare providers, in part to overcome pervasive perceptions that marginalised sex, gender, sexuality or intersex status do not exist or are not important in healthcare [5]. However, this can place an unreasonable burden on LGBTIQA+ individuals in healthcare or community settings that could be naïve, ignorant or openly hostile to their needs. Internationally, momentum has been building to shift this responsibility *to* healthcare providers and systems to make themselves inclusive and accessible to all [6]. 

We use the ‘person first’ term ‘people with disability’ in line with its use by advocacy and representative organisations, including People with Disability Australia and Women with Disability Australia. We note people may identify as having multiple disabilities, the phrase ‘people with disability’ aims to emphasise the disabling consequences of social structures and institutions. LGBTIQA+ people with disability have received very little attention, even within LGBTIQA+ inclusive practice movements. Emerging research has identified specific problems in accessing inclusive healthcare and disability support, in part due to the tendency in disability support to ignore the sexuality of people with disability [7,8]. As a result, many LGBTIQA+ people with disability find it very difficult or impossible to access the specific supports they need within disability services [9]. Our research focuses on how LGBTIQA+ people with disability experience healthcare and community services. We apply our findings to recommendations for the improvement of supportive services via critically approaching intersections of sex, gender, sexual identity and disability in relation to access.

## 2. Literature

### 2.1. Health Outcomes for LGBTIQA+ People with Disability

Research suggests a strong link between holding a marginalised sex, gender and/or sexuality identity or intersex status and experiences of abuse, poor health outcomes and acquired disability. For some people this link is strengthened by their specific disability and/or identity. People with disability are more likely to have poorer overall physical and mental health than people without disability [10], while people with intellectual disability have lower life expectancy and their rate of avoidable deaths is more than twice that of the general population [11,12]. Lesbian, gay and bisexual people have increased likelihood of disability, poor mental health outcomes, and substance use than their heterosexual counterparts [13]. For people with Autism Spectrum Disorder, the presumption that gender identities exist as an autistic trait or indeed as a ‘sequel to autism’ can be detrimental to both health outcomes and the ability to access care [14]. Research on LGBTI ageing demonstrates the cumulative effects of this marginalisation over the lifecourse, with older LGBTI people experiencing higher rates of disability, depression, anxiety and loneliness than the general community, as well as less social support [2]. People with intersex variations may be coerced into medical interventions to normalise sex characteristics in ways that cause harm, especially regarding sexuality and sexual health [15]. The effects of iatrogenic trauma and ongoing stigma related to intersex sex characteristics also produce poorer health outcomes for people with intersex variations [16]. For trans and gender diverse people, the classification of their identity as a mental disorder (currently ‘Gender Dysphoria’) and the ongoing impacts of this pathologisation can also produce an antagonistic relationship with medical professionals, and a reluctance to access health and other social services [17,18]. These are important differences to take note of between groups within the LGBTIQA+ umbrella, however, as we outline below, we purposefully did not require participants to disclose their specific attachment to these identity categories for this project. Indeed, our study had a broader scope, enabling people to think about common and shared experiences of health service use framed by solidarity within a connection to both LGBTIQA+ and disability identities and communities. 

Historical oppression and social inequalities are key factors influencing the experiences and lowered health outcomes of both LGBTIQA+ people and people with disability. For LGBTIQA+ people with disability, experiences of discrimination and oppression are compounded by intersecting, marginalised experiences and social identities, leading to multiple minority stresses [19]. ‘Minority stress’ refers to how marginalised groups experience stress that arises from experiences of stigma and discrimination, which leads to increased negative physical and mental health and social wellbeing outcomes [20]. Social support and networks have been shown to be protective factors against poor general health, disability and depression among lesbian, gay and bisexual people [21]. Yet, LGBTIQA+ people and people with disability experience higher levels of social exclusion across a range of settings, including healthcare and community services as well as schools, workplaces, and social events [22,23,24,25]. In addition, LGBTIQA+ people with disability may also experience ableist (disability-related) discrimination from within LGBTIQA+ services and communities and queerphobia (LGBTIQA+-related discrimination) from within disability services and communities, compounding experiences of social marginality and isolation [9,26]. Discrimination can also lead to internalised stigma and victimisation, which have been shown to be predictors of disability and depression among queer people [21]. Importantly, this is not inevitable. Indeed, as participants described in this study, pride in their LGBTIQA+ and disability identities facilitated strengthening and sustaining their psychological health in the face of discrimination. 

A number of systemic barriers impact on access to, and utilisation of, services by LGBTIQA+ people with disability. In particular, discriminatory and stigmatising attitudes towards LGBTIQA+ and/or people with disability are socially normalised and often held by those people working in the health, social and disability sectors [27,28,29]. Similarly, professionals often lack the knowledge, skills and confidence to deliver appropriate and responsive services to LGBTIQA+ people with disability, and tend not to be provided with adequate training, resources and other supports to improve their practice [9]. In part, this may well be because sufficient and appropriate education on the needs and experiences of LGBTIQA+ people with disability is simply not yet available. 

There is a clear need for a greater level of understanding of access and accessibility across all services, communities and agencies in order to engage effectively and respectfully with LGBTIQA+ people with disability. To our knowledge, this is the first Australian study that seeks to understand the health service needs and accessibility experiences of LGBTIQA+ people with disability, and to seek their input on the improvement of services, which is urgently needed.

### 2.2. Australia’s National Disability Insurance Scheme

In late 2019 a review of Australia’s National Disability Insurance Scheme (NDIS) legislation examined participants’ experiences of the NDIS and opportunities to improve systems and process. The review recommended that any amendment to NDIS legislation (NDIS Act [Cth] 2013) should include amendments to the principles of the NDIS Act to acknowledge the unique experiences of LGBTIQA+ people with disability, as agreed previously by Council of Australian Governments (COAG) in 2016 [30]. Despite acknowledging that experiences of discrimination and social exclusion are significant barriers to people accessing and navigating the NDIS, LGBTIQA+ people have not yet been identified as a priority community for assertive outreach or enhanced access support [30].

The NDIS Quality and Safeguards Commission is responsible for the registration and regulation of NDIS service providers. While a number of the NDIS Practice Standards are implicitly relevant and applicable to LGBTIQA+ people with disability, there is a lack of meaningful explicit reference to our rights. There is also a conspicuous lack of specific reference to ‘LGBTIQA+’, ‘queer’, ‘sexual orientation’, ‘gender identity’ or ‘intersex status’ in any of the NDIS Practice Standards and supporting guidelines. Despite references to LGBTIQA+ and the rights of people with disability across a range of Australian regulatory frameworks and laws (including equal opportunity and anti-discrimination legislation, Disability services legislation, and the Victorian Charter of Human Rights and Responsibilities), there are no statements that combine LGBTIQA+ and disability in any of these frameworks or laws. In this way, the specific access and health needs of LGBTIQA+ people with disability fail to be taken into account [31]. 

## 3. Methodology 

### 3.1. Participatory Action Research (PAR) and Employing Peer Researchers

We built on a foundation of Participatory Action Research (PAR) to frame and shape this study. Having a focus on “experiential, presentational, propositional and practical knowing” [32] (p. 325), was critical in how we engaged people who identified as being within the LGBTIQA+ spectrum and as a person with disability. LGBTIQA+ people with disability were present in the research as members of the Research Advisory Group (RAG), peer researchers (we use the term ‘peer researchers’ to describe LGBTIQA+ people with disability in this role to capture the significance placed on shared experience), and research participants. Peer researchers, commonly known also as ‘community researchers’, participated in all parts of this project, including the instigation of ideas, project planning, data collection and analysis, and contributing to and reviewing outputs such as this article. Propositional knowing of peer researchers related to formal theoretical knowledge of academic researchers and was practiced in this study in part by all researchers being seen as co-researchers; sharing responsibilities for action-focused knowledge development throughout the study. Such work connects with principles of citizenship seen in intellectual disability research [33,34]. This research used an iterative and action-focussed approach across all stages: the RAG met four times to oversee and guide the project, peer and academic researchers planned and completed data collection in four focus groups together, and engaged in two data analysis workshops conducted across two sessions to develop the themes and findings detailed in this article. This article is co-authored by members of the RAG, peer and academic researchers. Strong feedback loops in the research ensured our ideas were being co-developed throughout the project via the RAG and informed subsequent action research cycles of the overall project [35]. Deakin University Human Research Ethics Committee provided approval for the project (2019-207).

This study aimed to effect change within LGBTIQA+ and disability research by facilitating a critical-consciousness raising, encouraging analysis of social structures that form barriers to LGBTQIA+ people with disability, and actively attempting to subvert power dynamics that exist in more traditional research on sex, gender, sexuality, intersex, access, inclusion, and disability [36]. All researchers were dedicated to creating meaningful participation for LGBTIQA+ people with disability. This included spending time together to learn each other’s strengths, how we work, and how we could support each other throughout the project. We also acknowledged our shared or overlapping LGBTIQA+ and disability identities and experiences. Our research style contests the usual practice of producing people with disability especially as objects of research, and instead worked together to make roles and processes as inclusive and accessible as possible. We drew on work in inclusive disability research which has led the way in applying these practices for people with disability, particularly people with intellectual disability [37,38]. Together, these approaches represent a cohesive range of theoretical orientations and methods that “promote pluralism and creativity in the art of discovering the world and making it better at the same time” [39] (p. 3). 

We recognise that there are important differences experienced by different kinds of disability, as well as sex, gender, sexuality and intersex status. We note here that this study involved people from across the LGBTIQA+ spectrum, and included people who identified with various sensory, physical, and intellectual disability, as well as neurodiversity, acquired brain injury and complex communication needs. We did not ask participants to define or delineate their experience/s of disability, or their identification/s within the LGBTIQA+ umbrella. The main reason we made this decision was political: we know that people with disability are often required to explain their disability and its effects, to their own detriment, and that people who identify within the acronym LGBTIQA+ can feel pressured to justify their inclusion within a particular category. We sought to avoid these pressures, and instead focused our limited time together around experiences of health and community services and supports. This methodological choice was appreciated by participants and also protects their confidentiality, as some may have been able to be identified if we had required or included further levels of detail. 

The entire research team felt strongly that participant identification as an LGBTIQA+ person with disability was valid and sufficient. We note the same view of self-identification is taken by First Peoples Disability Network Australia in their work on community-driven research [40]. This approach was subsequently supported by the concerns expressed by participants, as we discuss below. We hope this project makes clear that there is a need for far more research into the experiences of LGBTIQA+ people with disability that focuses on specific identities, experiences and how they intersect. We hope this project will precipitate funding and pursuing such projects in the future, and we hope the approach to peer research we have taken will act as a framework for future research. 

### 3.2. Data Collection

Four focus groups with a total of 29 participants were held over three months in 2019, recruited online through social media, email and through personal networks of the research team. Recruitment was iterative, with additional guidance from the Research Advisory Group. Participants were people aged 18 years or over and self-identified as ‘LGBTIQA+ people with disability’. There were no additional or more specific eligibility criteria. Two focus groups were held in Melbourne (a large capital city), and one focus group was held in a regional Victorian city, which were conducted in spoken English with Australian Sign Language (Auslan) interpreters. One focus group was held for Deaf participants and conducted in Auslan in recognition of the connection to the Deaf community made possible by Deaf peer researcher (Sherrie Beaver) and academic researcher (O’Shea: a fluent Auslan user and interpreter). Drawing on the creativity and flexibility afforded within an inclusive PAR methodology, focus groups were coordinated by peer researchers with the support of academic researchers and invited people with any experience of disability, including Auslan users. 

Peer researchers worked in pairs to plan focus groups: identifying the location, venue, date and time, catering and in deciding if/when they would like support from the academic researchers before, during and after the focus group. Peer researchers advised on access needs including the identification of suitable Auslan-English interpreters, accessible venues and transit routes, making available a ‘quiet room’ for if/when participants needed a break, and the use of a ‘talking stick’ to ensure all participants were able to contribute. Academic researchers managed the consent process and operating digital voice recorders used for producing transcripts. Ethical and safety considerations were also addressed in line with advice from the Research Advisory Group, and included how to support participants who became distressed, and how to maintain confidentiality. 

Peer and academic researchers together developed a set of guidelines for running the focus groups. The guidelines provided a rundown of events: Acknowledgement of Country, explaining and collecting consent forms, introductions, and a list of potential interview questions and topics. The guidelines also included notes for various scenarios, such as what to do if group discussion went ‘off track’, if someone arrived late to the group, or if discussion stalled. The guidelines included a suggested list of themes for discussion, such as employment, housing, finances, relationships, services, and disability/LGBTQIA+ communities. All researchers collectively wrote the research questions using an open style that allowed participants to guide the discussion and share the issues of most significance to them. The first question was ‘what brought you here today?’ which was followed by open ended questions such as ‘what do you think is the most important thing we need to know?’ and ending with ‘is there anything else you’d like to tell us?’ 

Focus groups were digitally recorded for the production of typed transcripts. Participants were provided with a pen and paper if they wanted to make notes or write any follow up reflections to share with the research team. Some participants and some peer researchers chose to follow up their contribution in writing, which was included in the analysis process described below. 

### 3.3. Data Analysis

Qualitative data analysis of focus group transcripts and follow up contributions was conducted by the research team using thematic analysis and iterative categorisation [41,42] across three cycles of analysis, described below. The process was designed to facilitate a collaborative development of meaning and the analytic process of progressive focusing [43]. After the focus groups were professionally transcribed, academic researchers, following a thematic analysis approach, produced the initial analysis of emergent themes and cleaned the transcripts. Accessible summaries of emergent themes, as well as the full transcripts, were sent to peer researchers and the RAG, who shared their responses. The four peer researchers each worked with one of the overarching themes and prepared comments for the next cycle of analysis. Prompt questions were shared with peer researchers to guide in developing analysis skills, which suggested possible approaches, such as: explain the theme to others, give some examples from the focus group, and reflect on how this theme came out in the context of the focus group. 

The second cycle of analysis consisted of a half-day workshop with academic and peer researchers discussing our reading and responses to the themes and transcripts, and reflecting on each other’s contributions. Notes from this workshop were presented as bullet points and circulated to the research team. Following this workshop, the research team revisited the data by reflecting on our discussions, and refining or expanding the identified themes through a process of collaborative iterative categorisation [42]. Iterative categorisation allows researchers to “code and analyse their data by topic, event, story, verbal interaction, signifier, feeling, idea, category, theme, concept or theory, etc” [42] (p. 1096). 

The third cycle of analysis took place as the research team met for a second half-day workshop to discuss our reflections. Peer researchers identified the highest priority issues stemming from refining the emergent themes and this guided our way forward in producing resources from this project, including this article. As noted earlier, we purposefully did not differentiate the data analysis through individual identity categories.

## 4. Results and Discussion

Two main themes emerged from our data analysis: the difficulties of managing multiple identities when trying to access services, and community supports that produced pride, connection and solidarity, as well as practical information. Difficulties in managing multiple identities when attempting to access services was an overwhelming theme apparent in all focus groups. Participants talked about feeling ‘split’ between different parts of themselves, the stress of constantly coming out in different ways to different people, and the difficulties of making decisions about when, how and to whom they could disclose information to, and when they needed to mask parts of themselves in order to feel safe and/or receive services. Participants reported the importance of community-led services and especially supportive environments that they had found online, which could create a sense of pride and purpose that instilled confidence in accessing services. Yet participants also described feeling excluded from both LGBTIQA+ communities and disability communities that each failed to recognise LGBTIQA+ people with disability as part of these communities, with meaningful and important connections and contributions to make.

In order to preserve the anonymity of participants in this small study with a highly minoritised group of people, we cannot include identifiers on quotes provided in this article [35]. 

### 4.1. Managing Multiple Identities in Practice

Our research approach gave participants the opportunity to describe many layers of identity. We conceptualise identity as ‘how I am, and how I am known’, noting there may be a discrepancy between those elements [44,45]. Focus groups began with academic and peer researchers introducing ourselves and outlining our connections to the ‘researcher’, ‘LGBTIQA+’ and ‘disability’ identities. Participants introduced themselves in many different ways. Some disclosed about their experience of disability in detail, making brief mention of their sexual orientation, while others focussed on their gender or sexual identity, preferred pronouns, cultural identity and/or history of difficulties in accessing support [35].

Participants commented frequently on how having multiple identities means having to ‘come out’ many times, in different ways, in many different contexts. As one participant noted:

*We don’t come out once in our life; most of us come out every day, and I have to keep doing that around my sexuality, but also around my disability. My disability is very non-visual; it’s in here [points to head], and it’s the things that I can do and the things that I can’t do… but it is difficult, in a workplace, to cover both of these [identities] at once.*

A number of participants expressed fears and concerns about not having their identities understood or respected when navigating and engaging with services and individual providers, and again described the stress of trying to ascertain whether it was safe to disclose.

*I have to go back to my gynaecologist soon, because it’s time for my next check, since the last one five years ago, and since then, I’ve explored my gender, and come out, and things like that; so I’m sort of wondering if there’s any point in disclosing my trans identity, or if I should just closet myself. Because is there any point in doing the work to educate them? Because it’s almost certain they won’t know already. Or is that energy better spent on just getting through the whole experience?*

This was a concern for participants when navigating specific disability services, where they were unsure if it was safe to discuss their sex, gender, sexuality or intersex status. Participants also described uncertainty and tension around trying to access LGBTIQA+ services or events.

*So if I want to get autistic services, I don’t really talk about sexuality; if I want to go into queer spaces, I can’t really talk about disability and access… there’s not an understanding; there’s not a whole lot of cross-education.*

For LGBTIQA+ people with disability, these concerns mean having to constantly decide what information to prioritise or share with services, weighing decisions about what they think a service provider needs to know about their identities against the potential physical and emotional toll of having to explain themselves.

*A disproportionate number of disabled people are on Newstart, rather than DSP* [Disability Support Pension], *unfortunately; and that can be a really difficult landscape to navigate, as both a queer person, and a trans person, and all that kind of stuff, as well as a disabled person; because I have to disclose all those parts of identity as well as my access needs, police that they’re being met, police that I’m being gendered correctly, all of that kind of jazz; which makes every other part of life more difficult.*

Newstart is an income support payment for jobseekers in Australia. The Disability Support Pension (DSP) is a significantly higher payment. Not all people with disability will receive the DSP, partly because it is materially more difficult to access, practically and in its conditions. Participants noted issues with the DSP, for example, that it is means tested, meaning that if they partner with someone of a certain income they risk losing this payment and thus the critical independence it provides.

A common discussion among participants was the concept of ‘masking’ and ‘closeting’ identities when accessing services. They noted that just as LGBTIQA+ people with disability have to come out and/or explain themselves in multiple ways, they may also need to disguise or keep hidden different parts of themselves in different contexts. At times this meant having to strategically decide which parts of themselves to reveal, and the stress of decision-making. When trying to access health and other services in order to receive appropriate care, participants thought being able to describe information about their LGBTIQA+ identity and disability was relevant to receiving appropriate care, but did not know whether it would be safe to do so.

*I think often it’s easier to be one or the other, and you don’t often get to be both. You either get to be the person with a disability, and you don’t always disclose, as others have said; or you get to be the gay person, but you don’t get your disability side of you actually acknowledged, or sort of… I don’t know. I think you often get split between the two, or between however many there are.*

In addition to concerns about their identities not being recognised and needs not being understood, participants also expressed fear of being discriminated against when accessing and engaging with services, and of providers not knowing what to do to support them appropriately. For some, fear of discrimination or receiving poor quality care in health services resulted in them avoiding or delaying engagement in a variety of settings.

*I want to start study soon… but I’m afraid for two reasons, because I don’t know what level of support I’ll have with access needs, and things like that, because they’re not very explicit about that before you enrol. And I’m also worried about if my trans identity will be accepted.*

Some participants emphasised the importance of supporting their capacity to decide when and how to *not* disclose parts of their identity. In the case of some disability groups, such as people with cognitive impairment, agency in disclosure of sexual identity is often incorrectly taken for a lack of understanding or knowledge [46]. Participants emphasised how LGBTIQA+ people with disability had fewer options depending on the intersection of minority identities they belonged to, and/or the kinds of health or access needs they experienced.

*Whenever I need to get medical care, or therapy, or anything like that. You need to sort of pick which part is most important immediately, because there’s almost never any option that covers all the bases at once.*

Participants also described the additional burden and stress associated with having to determine whether a service can meet their access needs and advocating to have these needs met, which often meant incurring the additional financial costs of receiving access supports. At times the burden of coming out repeatedly was the result of individual service providers not respecting or appropriately responding to particular needs, or of systems not having the capabilities to accurately record information, such as non-binary genders and pronouns. As one participant described:

*I recently tried coming out to my disability employment services provider, because they have really not known a lot, so I think they’ve tried to take note of what I said, but there is no place on their system where they can note my pronouns, or my actual gender, or my preferred name - my actual name that I use, rather than my birth name.*

Services labelling themselves as ‘LGBTIQA+ friendly’ was seen as useful, but participants described wariness about whether this was a marketing ploy with little substance. In particular, many participants described experiencing negative treatment at such services.

*I still have anxiety about sharing my sexuality, and I live in a sexually and gender diverse household; I’m the carer of someone else with disability, and when I look for services, I actively search to see if they are LGBTI friendly; and even when I do find that they say they are, if they treat you really badly or discriminate based on the disability, I don’t then feel comfortable sharing the other part.*

This sentiment was shared by many participants, suggesting it can be difficult to present an integrated self via a set of identities and instead feeling forced to present only certain aspects of oneself in particular settings. This is important as the experiences of LGBTIQA+ people with disability shows how ‘access’ looks different for different people; pointing to the need for a holistic and comprehensive understanding of how access needs can be conceptualised and met [47]. As one participant put it:

*Knowing your accessibility needs can be really difficult sometimes, when disability is defined by what makes us frustrating for other people, as opposed to what our experiences of it actually are.*

### 4.2. Community Services and Supports 

‘Community’ is a somewhat disingenuous term as it implies a singular ‘disability community’ or ‘queer community.’ Rather, ‘community’ serves as shorthand for people who have in common particular identities. As with all groups of people, while they might share some experiences and values, there are many differences as well. In this context ‘community’ was identified variously connected to LGBTIQA+ and disability identities.

Many participants described experiencing mistreatment, not only via services, but also within their own communities. This included experiences of homophobia, biphobia and transphobia within disability communities, and ableism and ignorance in LGBTIQA+ communities. Participants described the despair that accompanied feeling like it was necessary to erase aspects of their identities across several communities. For many, this left them feeling excluded, marginalised and unsafe within their own communities, and this had a detrimental impact on their ability to participate in accessing health and other services they might want or need.

*I think there’s so many social barriers, in terms of people’s ableism and negative attitudes around disability, within the broader community, but also within the LGBTI community…there’s so many assumptions about people with disabilities, around our sexuality, our desire. Being viewed as not desirable or less than other people. You know, trying to find a relationship, but also trying to find a pash on a Saturday night is really hard.*

Failing to address these experiences of discrimination within both LGBTIQA+ and disability communities makes people who exist within both these worlds at risk of further isolation. LGBTIQA+ people with disability are often expected to either: tolerate these kinds of fragmentations of their identities in order to connect, or to take on the burden and responsibility of addressing these marginalisations and exclusions themselves, which they are actively experiencing. Neither of these ‘choices’ recognise the systemic issues of in/access as oppressive and instead continue to focus on individuals’ ability to be ‘included.’ 

This was a key point discussed in the focus groups, relating to difficulties in access and inclusion within community spaces, where competing accessibility needs or the values and aspirations of particular groups could clash. As one participant reported,

*Just because you’re disabled doesn’t mean that you’re inclusive to people with all different types of disability, and sometimes little cliques can form.*

Participants stressed the importance of understanding the breadth and variety of access needs, emphasising that ‘one access size’ does not fit all people. In our own experience of creating accessible spaces for the focus groups, we experienced some of these clashes. For example, we needed an environment which worked for people with sensory sensitivity (requiring dim lighting) and people with vision impairment (requiring bright lighting) [35]. Participants also drew attention to the complexity of psychosocial disability, where access needs could be harder to describe and less likely to be understood. Many participants lamented that terms such as ‘accessibility’ and ‘inclusion’ could be stickered onto events, organisations or services without being relevant to them. Participants described the implications of misusing of these terms:

‘Inclusive’ really isn’t inclusive yet. So a lot of places are being advertised as an inclusive event, yet they’ve not actually done any research in regards to finding out about disabilities and what inclusive changes need to happen for people with disabilities to access those services. So yeah, it kind of… it raises the question of going, “Okay, well, all of these things are being promoted as accessible for us, yet they’re not.”

In many cases participants saw this as evidence of a lack of understanding or consultation with people with disability. Participants acknowledged that efforts had been made by some LGBTIQA+ event organisers to improve some aspects of accessibility but highlighted the importance of ensuring that more community events and spaces are accessible to more people, taking into account physical, financial and social access barriers. In particular, they spoke of the need for organisers of LGBTIQA+ events to plan for and provide access for people with disability, make clear what those accessibility adjustments are, and the positive impact of doing so.

*I think actually the best example of accessibility within the LGBTI community I think has to go to [LGBTIQA+ cultural festival] Midsumma, because they are proactively starting to make that information available in events and booklets. So when you open up the booklet, they’re starting to include wheelchair access, they’ve got Auslan there, breakout spaces, and things like that.*

A prominent theme was the role of community in promoting a sense of belonging and acceptance, and the importance of peer connections for instilling pride and counteracting shame. For example, one participant described the positive impact of discovering their interpreter was also part of the queer community:

I got the vibes from them that they’re one of us, and I was sort of thinking, how long have they been part of the LBGTI community, and they said that they’re gay, so we had that camaraderie, and we were able to talk to each other about it, and I could add my sass, and I could hear him using that sort of language when he was interpreting for me, and I really loved that.

This experience of connection, solidarity and belonging directly influenced participants’ feelings about, and capacity to, seek support from services. Online communities were seen as particularly important for LGBTIQA+ people with disability, where they were able to find community-led, accessible information, especially for people who may not be able to connect in physical spaces. As one participant described:

First time I ever saw another LGBTI person with disability was actually on [dating app] Grindr. I’d never actually met anyone. I never actually knew there were other people out there; and when it started to show up on Grindr, that was when I was like, “Wow, there are actually other people out there.” Which is a really powerful thing.

Participants also recounted how opportunities to connect offline with peers can be limited for LGBTIQA+ people with disability and that this is compounded for people already experiencing social isolation, especially those with chronic mental illness, complex communication, or who live in regional and rural areas. As one participant put it:

You get to meet many people in the Deaf world, but out in regional areas, you’re the only one, essentially, so being a woman, living out there and being a lesbian, there’s not a lot of options for me out there. It’s very limiting.

Online spaces were also described in terms of providing people with security over what and how much they reveal about their identities. This provided a way to practice tailoring the kinds of information they felt comfortable sharing, and gaining acceptance through being open about parts of themselves that were positively received. This instilled a sense of pride and purpose that could translate into a stronger position of advocating for their needs and/or attempting to access services, as well as finding support in how to go about doing so.

*I find a lot of Facebook groups really helpful. There are really specific identity-based Facebook groups, and also ones that are specifically tailored to different political leanings and things like that, so you can feel accepted in multiple facets of identity at once. Those are all our own voices, community-based spaces - they’re not set up by other people. It’s all ourselves. *

Participants celebrated the chance to explore their pride in identity through online connections. Some participants reported feeling a sense of agency in making choices about which identities to make public or to express in each setting. Key to participants was the possibility of expressing agency as decision makers, where taking control of when, how, what and with whom to share was balanced with questions of safety. For some, a developing comfort and pride with their own identity made disclosure more possible.

*My experience, when I was younger, I wasn’t sure about my identity, I was really confused. I didn’t know – I just pushed that side of myself down. I didn’t want people to tease me, I didn’t like that. I put a mask on, straight away. I wasn’t comfortable with who I was. But later, when I was older, I saw in my Deaf community that I was accepted. You know, why am I so worried about it. Why was I pretending to be straight?*

Participants discussed the role of building resilience and capacities within communities, which could also act as an important source of relevant, tailored information, including about specific disability and LGBTIQA+ identities, navigating difficult healthcare or bureaucratic systems, and which healthcare providers or services had been useful to others. Yet participants also emphasised that they felt they were made responsible for their own access needs, both in health services and community events and organisations, and that this is inherently discriminating. This was also repeatedly described as exhausting, and participants called for services and organisations to reach out, beyond simply recognising that LGBTIQA+ people with disability exist, towards actively supporting their needs and capacities to participate. 

## 5. Conclusions

The key finding from this research is that the variety of identities LGBTIQA+ people with disability hold tend to be critically misunderstood in services they rely on, including health services, disability services, LGBTIQA+ services, and in these communities. While some participants felt able to present their ‘whole self’ in different settings, or found comfort with selective or partial disclosure, many people expressed discomfort, frustration, and despair at having to ‘wear a heterosexual, cisgender mask’ in disability services, and of feeling excluded from LGBTIQA+ spaces that they could on access or that did not recognise their experience of disability. We might have expected difficulty with disclosure of LGBTIQA+ identities in disability services, yet we also found difficulty with expressing disability identities within LGBTIQA+ communities and services. If people are not able to confidently and safely access healthcare, and community services, then their right to receive appropriate healthcare and support is denied. 

The impact of *not* being able to disclose identities and status safely encroached on the home environment when it involved family, guardianship or home-based care. There are potential implications to treatment, health interventions or support services that are designed without full practitioner information, particularly in a medical setting. We note that the responsibility for such implications cannot and must not rest with the individual, and existing knowledge around misdiagnosis and diagnostic overshadowing bias [48] should be extended and applied to this group.

Our findings suggest that disability services often do not comprehend the ways their services are inaccessible and inappropriate to LGBTIQA+ people. Conversely, LGBTIQA+ services often fail to provide services that are accessible and appropriate to people with disability. A failure to understand multiple identities in health and social care settings is a major restriction to access and healthcare. These are integrated issues which must be addressed as such. Accordingly, centring the experiences of LGBTIQA+ people with disability in reforms will focus the development of inclusive practices that benefit more LGBTIQA+ people and people with disability as well. 

The study was limited by several factors. Firstly, the project scope was to focus on disability as a whole, rather than to focus on particular kinds of disability, and to include anyone who identified within the LGBTIQA+ spectrum. Obvious and important differences exist and were borne out in the focus groups, which shows how much further research into specific disabilities and intersections with particular LGBTIQA+ identities is needed. Since data collection was completed, Victorians have experienced restrictions related to the global COVID-19 pandemic. Much research has been moved online, and we have heard early anecdotal reports that for some people with disability, online participation is more accessible (for example, through better capacity to manage fatigue and reducing travel time). All focus groups were run in person, adding an online focus group option would have increased the opportunities for participants from regional areas and those for whom it provided a greater level of access. 

Inclusive practice means a lot more than putting up a sticker or building a ramp. There are no easy, simple solutions to the complex ways in which LGBTIQA+ people with disability experience social exclusion and marginalisation, including in using or trying to access a host of health and advocacy services. Meaningful inclusion means that, from design to delivery, LGBTIQA+ people with disability work at all levels of planning and management, in ways which value our expertise and commit to outcomes that offer meaningful transformations in policy and practice to LGBTIQA+ people with disability. As demonstrated in this research, people for whom access and accessibility is being planned must be included in the processes of consultation, research, service and policy development. This may well mean actively seeking out LGBTIQA+ people with disability and facilitating our involvement across a wide range of roles, including as staff, volunteers (with a note that people with disability deserve to be paid appropriately), designers, board members, spokespeople, and more. ‘Access’ must be conceptualised more holistically, as we have argued in this article. The participants in this study clearly wanted to be actively involved in making solutions, including being asked about their needs and experiences for the purpose of improving services. In this way, we can see how research too must be made accessible and include meaningful participation of research subjects, such as co-publishing accessible outputs available with open access. 
